# Diagnostic performance of ultrasound in acute cholecystitis: a systematic review and meta-analysis

**DOI:** 10.1186/s13017-023-00524-5

**Published:** 2023-11-30

**Authors:** Sih-Shiang Huang, Kai-Wei Lin, Kao-Lang Liu, Yao-Ming Wu, Wan-Ching Lien, Hsiu-Po Wang

**Affiliations:** 1 Department of Emergency Medicine, National Taiwan University Hospital and College of Medicine, National Taiwan University, No.7, Chung-Shan South Road, Taipei, 100 Taiwan; 2 Department of Medical Imaging, National Taiwan University Cancer Center, National Taiwan University Hospital, Taipei, Taiwan; 3 Department of Medical Imaging, College of Medicine, National Taiwan University, Taipei, Taiwan; 4 Department of Surgery, College of Medicine, National Taiwan University, Taipei, Taiwan; 5 Department of Emergency Medicine, College of Medicine, National Taiwan University, Taipei, Taiwan; 6 Department of Internal Medicine, College of Medicine, National Taiwan University, Taipei, Taiwan

**Keywords:** Acute cholecystitis, Ultrasound, Emergency physician

## Abstract

**Background:**

An updated overview of ultrasound (US) for diagnosis of acute cholecystitis (AC) remains lacking. This systematic review was conducted to evaluate the diagnostic performance of US for AC.

**Methods:**

A systematic review was conducted following PRISMA guidelines. We meticulously screened articles from MEDLINE, Embase, and the Cochrane Library, spanning from inception to August 2023. We employed the search strategy combining the keywords "bedside US", "emergency US" or "point-of-care US" with "AC". Two reviewers independently screened the titles and abstracts of the retrieved articles to identify suitable studies. The inclusion criteria encompassed articles investigating the diagnostic performance of US for AC. Data regarding diagnostic performance, sonographers, and sonographic findings including the presence of gallstone, gallbladder (GB) wall thickness, peri-GB fluid, or sonographic Murphy sign were extracted, and a meta-analysis was executed. Case reports, editorials, and review articles were excluded, as well as studies focused on acalculous cholecystitis. The study quality was assessed with the Quality Assessment of Diagnostic Accuracy Studies-2 (QUADAS-2) tool.

**Results:**

Forty studies with 8,652 patients were included. The majority of studies had a low risk of bias and applicability concerns. US had a pooled sensitivity of 71% (95% CI, 69–72%), a specificity of 85% (95% CI, 84–86%), and an accuracy of 0.83 (95% CI, 0.82–0.83) for the diagnosis of AC. The pooled sensitivity and specificity were 71% (95% CI, 67–74%) and 92% (95% CI, 90–93%) performed by emergency physicians (EPs), 79% (95% CI, 71–85%) and 76% (95% CI, 69–81%) performed by surgeons, and 68% (95% CI 66–71%) and 87% (95% CI, 86–88%) performed by radiologists, respectively. There were no statistically significant differences among the three groups.

**Conclusion:**

US is a good imaging modality for the diagnosis of AC. EP-performed US has a similar diagnostic performance to radiologist-performed US. Further investigations would be needed to investigate the impact of US on expediting the management process and improving patient-centered outcomes.

**Supplementary Information:**

The online version contains supplementary material available at 10.1186/s13017-023-00524-5.

## Introduction

Acute cholecystitis (AC) is one of the most common diseases in emergency departments (EDs), occurring in 3–10% of patients with acute abdominal pain [1]. It generally results from cystic duct obstruction by a gallstone, followed by inflammation of the gallbladder (GB) [2].

Diagnostic imaging modalities for AC include ultrasound (US), computed tomography (CT), or hepatobiliary iminodiacetic acid (HIDA) scan [3]. A 2012 meta-analysis reported that the HIDA scan had the highest diagnostic accuracy for AC [4]. However, US has non-radiating, easily accessible, and inexpensive characteristics, becoming the first-line diagnostic tool in emergency settings. In recent years, there has been a significant increase in the number of publications regarding the use of US for the diagnosis of AC. Up-to-date evidence is still lacking. Further, US is performed by radiologists traditionally. It is unclear whether the diagnostic performance differs when performed by other sonographers such as emergency physicians (EPs) or surgeons.

Hence, we aim to perform a meta-analysis to investigate the diagnostic performance of US for AC.

## Methods

This meta-analysis adhered to the Preferred Reporting Items for a Systematic Review and Meta-analysis of Diagnostic Test Accuracy Studies (PRISMA-DTA) Statement [5]. The meta-analysis protocol was registered in PROSPERO (CRD42023425075). The ethical committee review was waived at the study institution.

### Search strategy and study selection

To identify relevant articles for our study, we conducted a comprehensive search in three databases: MEDLINE, Embase, and Cochrane Library. The search included articles published before August 2023, without any language restrictions. We employed the search strategy combining the keywords "bedside US", "emergency US" or "point-of-care US" with "AC". Two reviewers (SSH and KWL) independently screened the titles and abstracts of the retrieved articles to identify suitable studies. The inclusion criteria encompassed articles investigating the diagnostic performance of US for AC. We excluded case reports, case series, editorials, and review articles from our search strategy, as well as studies focused on acalculous cholecystitis. The complete literature search strategy is available in Additional file 5: Table S1.

### Data extraction and quality assessment

The quality of the included studies was evaluated by two independent reviewers (SSH and KWL) using the Quality Assessment of Diagnostic Accuracy Studies-2 (QUADAS-2) tool [6]. Any discrepancies between the reviewers were resolved through discussion involving a third author (WCL).

### Data synthesis and analysis

We extracted and summarized data from each study into 2 × 2 contingency tables to perform sensitivity and specificity analysis. To mitigate bias in the presence of zero observations in false-positive or false-negative results, we applied a continuity correction of 0.5. Summary estimates of sensitivity, specificity, predictive values, likelihood ratios, and accuracy along with their 95% confidence intervals (CIs) were calculated using a bivariate random-effects model with restricted maximum likelihood estimation for diagnostic meta-analysis [7]. The forest plot was used to visually represent the pooled summary estimates and their 95% CIs.

Additionally, we performed a subgroup analysis to assess the diagnostic performance of US among different sonographers, namely EPs, surgeons, and radiologists. Furthermore, we conducted a separate subgroup analysis to investigate the diagnostic accuracy of various sonographic findings in diagnosing AC.

To measure heterogeneity between the included studies, we utilized the inconsistency index *I*^2^. Additionally, we assessed publication bias using Deek’s test [8]. Statistical significance was defined as a *p* value < 0.05. All analyses were conducted using R software version 4.3.0 (R Foundation for Statistical Computing, Vienna, Australia).

## Results

**Fig. 1:**
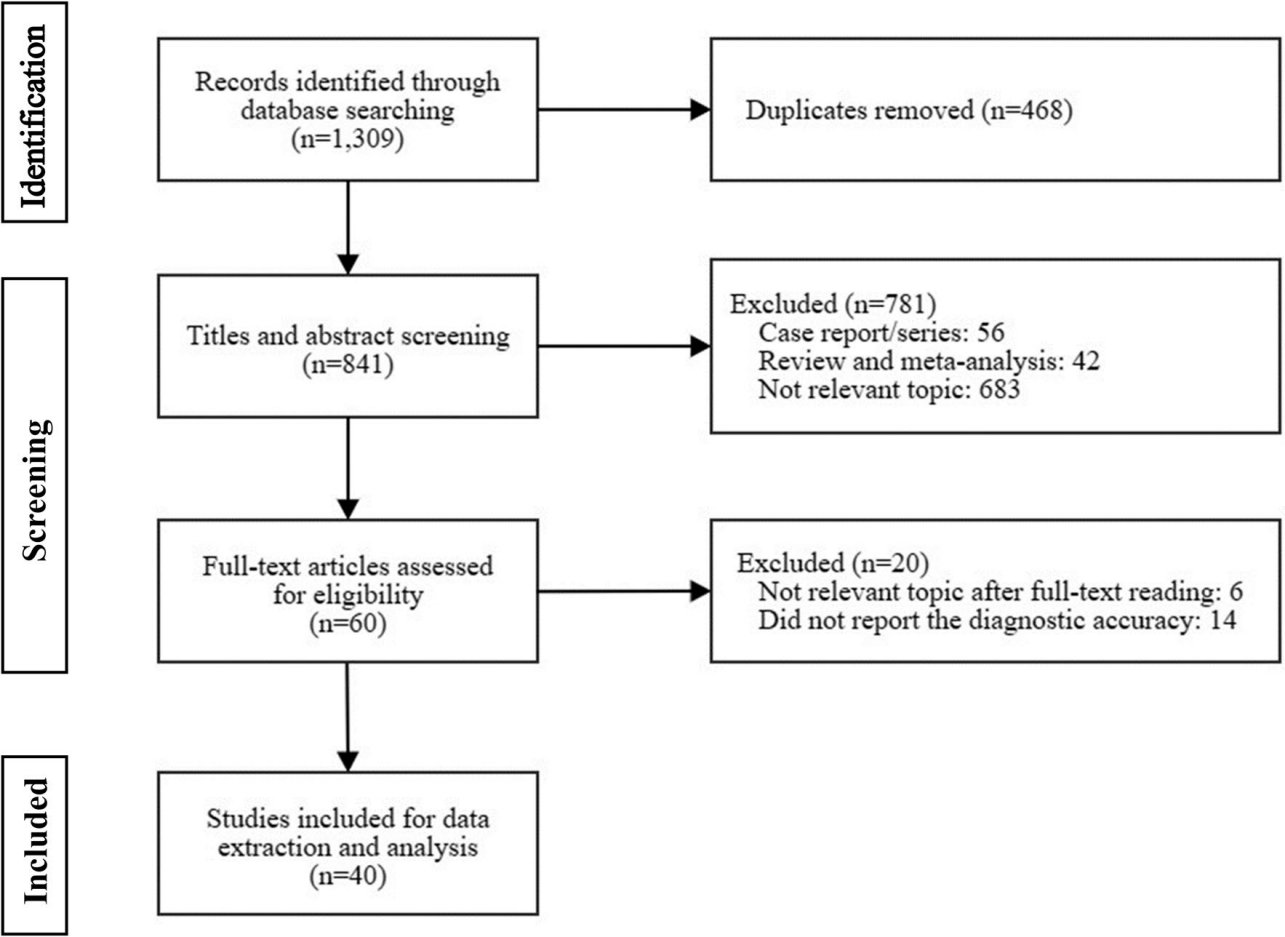
The Preferred Reporting Items for Systematic Reviews and Meta-Analyses (PRISMA) diagram

Figure 1 depicts a flowchart that outlines the inclusion and exclusion process. A total of 1309 studies were identified through MEDLINE, Embase, Cochrane Library, and manual searches of the reference list of the included articles. After the initial screening and removal of duplicates, 60 studies were left for full-text article review. Among them, 20 studies were excluded during the full-text review as they did not present relevant findings on the topic or report the diagnostic accuracy of US. Consequently, 40 studies were included for data extraction and meta-analysis [9–48]. We also generated a summary receiver operating characteristics (SROC) curve to assess the performance of US in detecting AC (Additional file 1: Fig. S1).

### Quality of the included studies

**Fig. 2:**
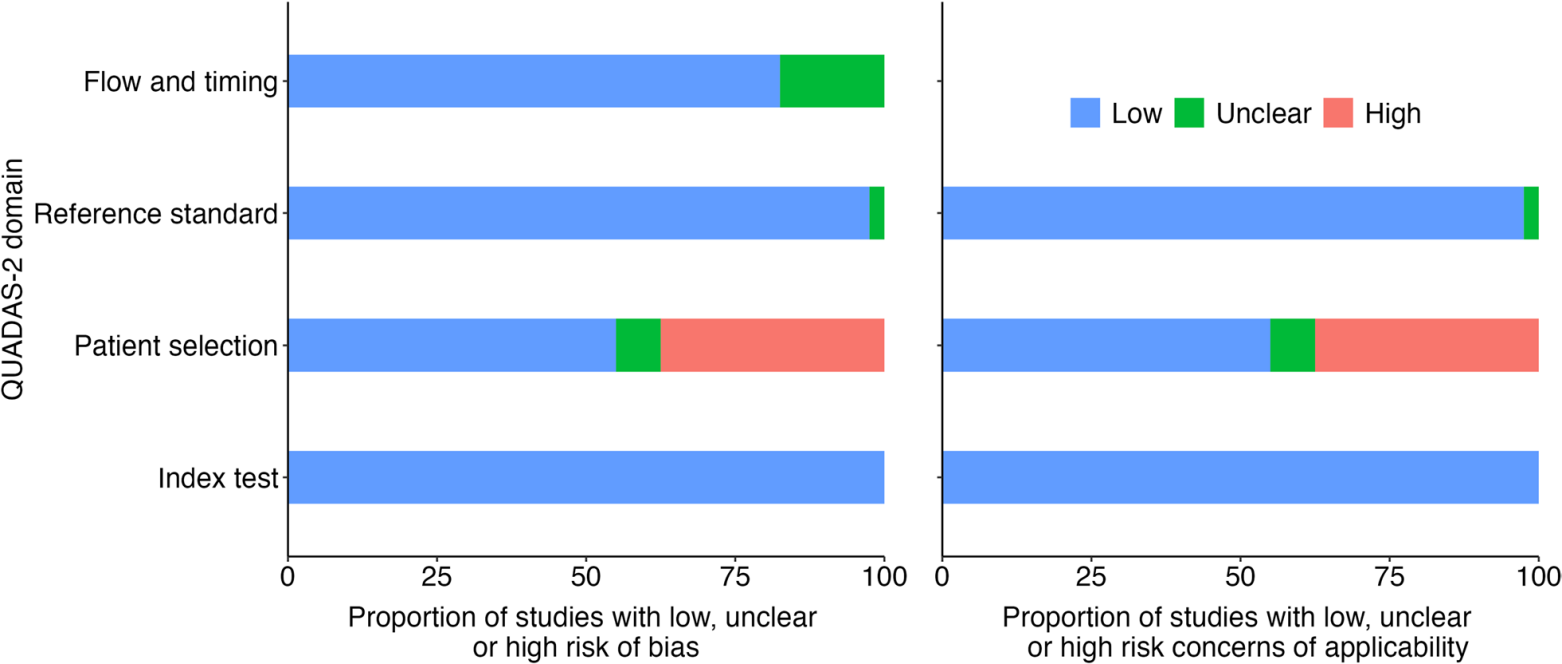
The summary of the quality assessment results of the included studies

Figure 2 shows the risk of bias and applicability of the included studies. In the risk of bias assessment, the majority of studies, except for patient selection, had a low risk of bias across four domains. However, more than 25% of the studies were identified as having a high risk of bias due to their utilization of a case–control study design and their non-consecutive or non-random enrollment of sample patients. Turning to applicability, most studies received a low-risk score in the reference standard and index test domains. Nevertheless, some studies were deemed to have a high risk of bias in terms of applicability because they exclusively focused on post-cholecystectomy patients instead of a more diverse and representative patient cohort.

### Diagnostic performance of US

**Fig. 3:**
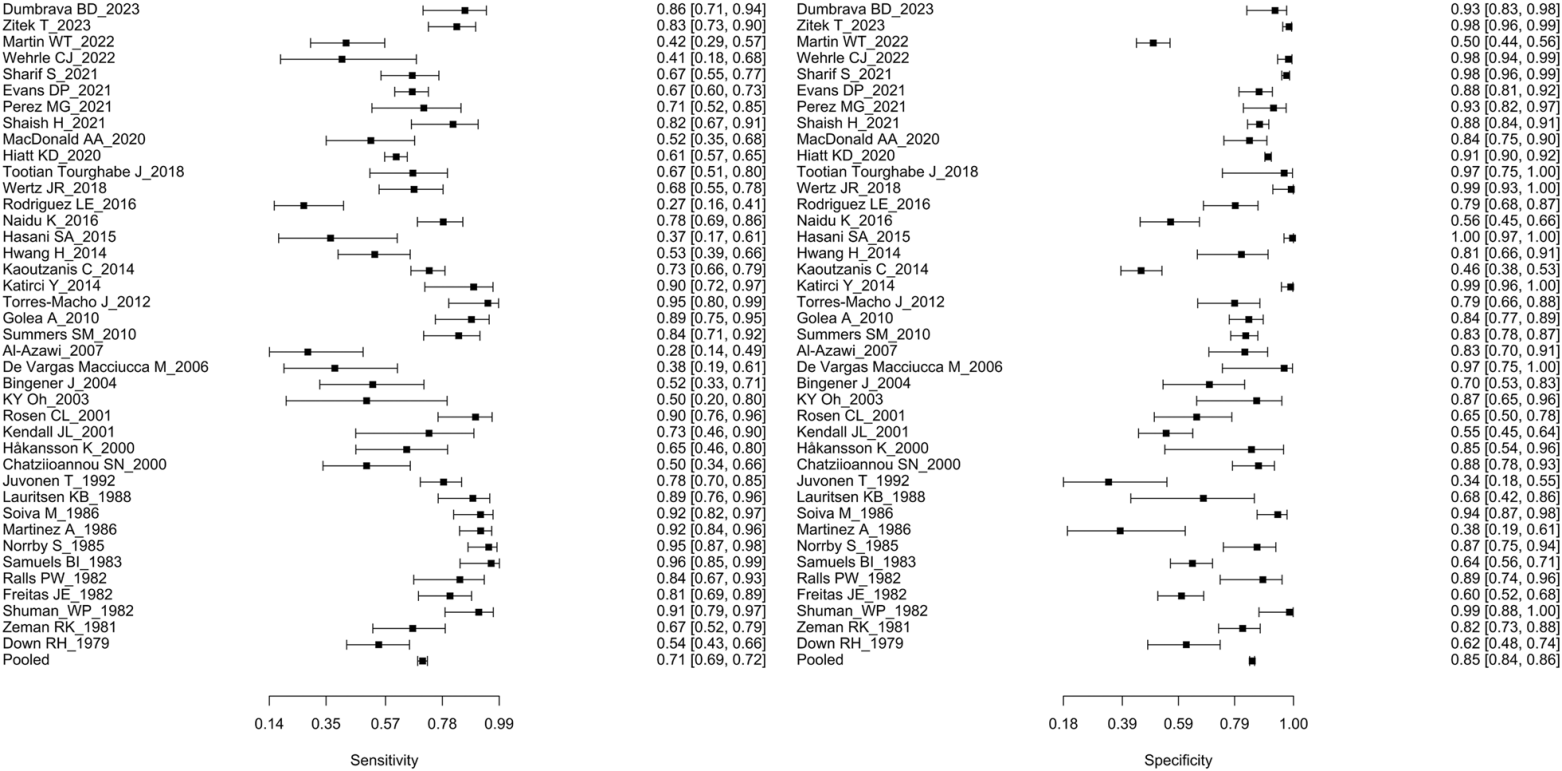
The forest plot of diagnostic performance of ultrasound for the diagnosis of acute cholecystitis

Across all 40 studies, a total of 8652 patients were included, with an average age of 45.9 years and a male gender composition of 34%. Detailed information about the included studies can be found in Table 1. The overall sensitivity was 71% (95% CI, 69–72%), while the specificity was 85% (95% CI, 84–86%) (Table 2, Fig. 3). The positive likelihood ratio (PLR) was 4.80 (95% CI, 3.33–6.78), and the negative likelihood ratio (NLR) was 0.33 (95% CI, 0.25–0.41). The accuracy was 0.83 (95% CI, 0.82–0.83), demonstrating good diagnostic performance. Heterogeneity among the studies was high (*I*^2^ = 89.7%; 95% CI, 87–92%), which could be due to varying patient enrollment criteria and the presence of potential confounders due to non-randomized assignment in the included studies. No significant publication bias was detected through Deek’s test (*p* value = 0.39).

**Table 1:** Detailed information on the included studies

References	Year	Country	Included patients	Age	Male (%)	Sonographer	Reference standard
Dumbrava et al. [9]	2023	Ireland, Portugal & Italy	84	50.5	34	Surgeon	RadUS
Zitek et al. [10]	2023	USA	348	48.0	40	Emergency physician	Pathology, HIDA, CT, RadUS
Martin et al. [11]	2022	USA	308	40.3	27	NR*	Pathology
Wehrle et al. [12]	2022	USA	147	37.8	28	Emergency physician	Pathology
Sharif et al. [13]	2021	Canada	577	NR*	NR*	Emergency physician	Pathology, laparoscopy, RadUS, CT
Evans et al. [14]	2021	USA	332	NR*	NR*	Emergency physician	RadUS, final diagnosis
Perez et al.[15]	2021	USA	73	49.7	33	Radiologist	Pathology, PTC fluid, cholangiography
Shaish et al.[16]	2021	USA	319	48	23	Radiologist	Pathology, Clinical follow-up
MacDonald et al. [17]	2020	New Zealand	116	48.0	23	Emergency physician Surgeon	RadUS
Hiatt et al. [18]	2020	USA	2859	41.0	31	Radiologist	Pathology, clinical diagnosis
Tourghabe et al. [19]	2018	Iran	51	42.3	18	Emergency physician Radiologist	Pathology
Wertz et al.[20]	2018	USA	56	66.0	93	Radiologist	Pathology, PTC fluid, Clinical follow-up
Rodriguez et al. [21]	2016	USA	106	44.0	34	Radiologist	Pathology
Naidu et al. [22]	2016	Australia	169	43.0	36	Surgeon	Pathology
Hasani et al. [23]	2015	Iran	150	47.4	56	Emergency physician	Final diagnosis
Hwang et al. [24]	2014	Canada	83	55.5	36	Radiologist	Pathology
Kaoutzanis et al. [25]	2014	USA	360	49.4	34	Radiologist	Pathology
Katirci et al. [26]	2014	Turkey	168	51.7	35	Emergency physician	RadUS
Torres-Macho et al. [27]	2012	Spain	78	67.8	55	Emergency physician	RadUS
Golea et al. [28]	2010	Romania	179	59.3	38	Emergency physician	Pathology
Summers et al. [29]	2010	USA	277	36.0	27	Emergency physician Radiologist	Pathology, clinical follow-up
Al-Azawi et al. [30]	2007	Ireland	70	50.0	17	Radiologist	Pathology
Macciucca et al. [31]	2006	Italy	30	66.6	66	Emergency physician	Pathology
Bingener et al. [32]	2004	USA	55	37.0	15	Radiologist	Pathology
Oh et al.[33]	2003	USA	24	52.8	25	Radiologist	Pathology
Rosen et al. [34]	2001	USA	76	49.0	28	Emergency physician	Clinical follow-up
Kendall et al.[35]	2001	USA	109	39.0	21	Emergency physician	RadUS
Håkansson et al. [36]	2000	Sweden	35	56.0	51	Radiologist	Surgery, pathology
Chatziioannou et al. [37]	2000	USA	107	57.0	46	NR*	Pathology, clinical follow-up
Juvonen et al.[38]	1992	Finland	129	63.0	47	Radiologist	Pathology
Lauritsen et al. [39]	1988	Denmark	54	66.0	58	NR*	Pathology, clinical follow-up
Soiva et al. [40]	1986	Finland	135	56.0	39	Radiologist	Pathology, clinical follow-up
Martinez et al.[41]	1986	Spain	98	69.0	69	NR*	Pathology
Norrby et al.[42]	1985	Sweden	120	NR*	NR*	Radiologist	Pathology
Samuels et al. [43]	1983	USA	190	NR*	NR*	NR*	Pathology, clinical follow-up
Ralls et al. [44]	1982	USA	54	NR*	NR*	Radiologist	Pathology, clinical follow-up
Freitas et al. [45]	1982	USA	192	NR*	NR*	NR*	Pathology, clinical follow-up
Shuman et al. [46]	1982	USA	74	52.0	63	NR*	Pathology, clinical follow-up
Zeman et al. [47]	1981	USA	144	48.5	38	Radiologist	Pathology, clinical follow-up
Down et al. [48]	1979	Australia	116	46.1	32	NR*	Pathology

^*^RadUS, radiology ultrasound; NR, not reported; PTC, percutaneous cholecystostomy; HIDA, hepatobiliary iminodiacetic acid scan; CT, computed tomography

**Table 2:** The pooled estimates of diagnostic performance of ultrasound for acute cholecystitis

	No. of studies	Sensitivity (95% CI)	Specificity (95% CI)	PLR (95% CI)	NLR (95% CI)	Accuracy (95% CI)
Pooled	40	0.71 (0.69–0.72)	0.85 (0.84–0.86)	4.80 (3.33–6.78)	0.33 (0.25–0.41)	82.5 (81.9–83.0)
Emergency physician	14	0.71 (0.67–0.74)	0.92 (0.90–0.93)	10.40 (4.80–20.3)	0.32 (0.21–0.46)	84.3 (83.2–85.4)
Surgeon	3	0.79 (0.71–0.85)	0.76 (0.69–0.81)	5.51 (1.58–16.40)	0.28 (0.17–0.48)	83.4 (79.7–87.3)
Radiologist	18	0.68 (0.66–0.71)	0.87 (0.86–0.88)	4.50 (2.83–6.87)	0.35 (0.23–0.49)	77.4 (76.0–78.8)

*CI* Confidence interval; *PLR* Positive likelihood ratio; *NLR* Negative likelihood ratio

### Subgroup analysis for sonographers

The subgroup analysis included 14 studies involving EPs, 3 studies involving surgeons, and 18 studies involving radiologists. Two studies compared the performance between EPs and radiologists, and one compared EPs with surgeons, while 8 did not provide detailed information on the sonographers.

The pooled sensitivity and specificity of US were 71% (95% CI, 67–74%) and 92% (95% CI, 90–93%) performed by EPs, 79% (95% CI, 71–85%) and 76% (95% CI, 69–81%) performed by surgeons, and 68% (95% CI 66–71%) and 87% (95% CI, 86–88%) performed by radiologists, respectively (Additional files 2, 3 and 4: Figs. S2, S3 and S4 and Table 2). There were no statistically significant differences in sensitivity, specificity, PLR, NLR, and accuracy among the three groups.

### Subgroup analysis of sonographic findings

The sonographic findings and their relationship to the diagnosis of AC are summarized in Table 3. Notably, not all of the included studies provided detailed information regarding individual sonographic findings.

**Table 3:** The pooled estimates of the sonographic finding for the diagnosis of acute cholecystitis

Sonographic finding	No. of studies	Sensitivity (95% CI)	Specificity (95% CI)	PLR (95% CI)	NLR (95% CI)	Accuracy (95% CI)
GB wall thickness	8	0.45 (0.40–0.50)	0.84 (0.81–0.87)	3.29 (1.83–5.69)	0.52 (0.31–0.75)	59.0 (57.2–60.8)
Gallstone	7	0.88 (0.84–0.91)	0.71 (0.65–0.76)	4.10 (1.46–11.5)	0.16 (0.08–0.29)	93.2 (92.3–94.2)
Peri-GB fluid	7	0.26 (0.22–0.31)	0.92 (0.89–0.93)	5.62 (1.71–13.9)	0.72 (0.58–0.89)	63.9 (62.2–65.7)
Sonographic Murphy sign	7	0.51 (0.46–0.56)	0.75 (0.72–0.78)	3.6 (1.07–9.74)	0.58 (0.32–0.96)	65.9 (63.9–68.0)

*GB* Gallbladder; *CI* Confidence interval; *PLR* Positive likelihood ratio; *NLR* Negative likelihood ratio

## Discussion

We performed a systematic review and meta-analysis to investigate the diagnostic performance of US for AC. Forty studies with a total of 8,652 patients were included. To the best of our knowledge, this is the largest meta-analysis currently, providing updated evidence.

Our results revealed US had a sensitivity of 71%, a specificity of 85%, and an accuracy of 0.83, indicative of good discriminability. Also, the sensitivity and specificity were similar among those performed by EPs, surgeons, and radiologists. Further, the presence of gallstones had a higher sensitivity for AC. However, most of the studies used combinations of sonographic findings for the diagnosis of AC.

Clinical symptoms and signs of AC had varying sensitivity and specificity [49]. The Tokyo guidelines suggest using imaging studies such as US, CT, and HIDA scans for the diagnosis of AC, in conjunction with detailed history, complete clinical examination, and laboratory tests [3]. Although HIDA has excellent diagnostic performance for AC with a sensitivity and specificity above 90% [4], its utilization is limited in emergency practice due to the required resources, time, and exposure to radioactive isotopes [50]. By contrast, US is a valuable tool for its non-ionizing, low-cost, and easy-to-use characteristics. US is considered the first-line imaging modality in recently published guidelines for the diagnosis of AC [3, 50]. Our review provides the evidence that US is a good diagnostic tool with discriminative power.

The American College of Emergency Physicians states that US is an essential skill in emergency practice, and GB-US is included in 12 core applications [51, 52]. It also indicates that 25 sonographic examinations of GB should be performed as a minimum requirement for training and accreditation [51]. In recent years, US has broadly used and increased integration into emergency practice. There were also a rising number of studies regarding the EP-performed US.

In our review, the diagnostic performance was similar between EPs and radiologists. Half of the 14 studies that EPs performed US reported the training background [10, 12, 13, 23, 27, 29, 34]; however, the level of training could range from novices (the first-year residents) to attendings [10]. Summers et al. [29] reported an intraclass correlation coefficient of 0 (95% CI, 0–0.13), suggestive of similar performance at different levels. Although the inter-rater reliability was not thoroughly evaluated in the majority of the studies regarding the EP-performed US, EPs could achieve proficiency using US as a part of physical examination for the assessment of GB diseases [26].

Moreover, US also demonstrates time efficiency in several studies [9, 21, 25]. The mean time interval between the surgeon-performed US and the surgery was significantly lower than that between the radiologist-performed and surgery (2.3 vs. 11.9 h) [9]. Similar results were observed between those receiving radiologist-performed US and HIDA scans [21, 25]. However, evidence regarding the effect of EP-performed US in the fastening clinical management process or patient-centered outcomes (length of stay and mortality) of patients with AC is still lacking.

In our review, the presence of gallstones exhibited optimal performance for the diagnosis of AC. However, most of the included studies used the combination of the presence of gallstones with at least one additional inflammatory sign such as GB wall thickness, peri-GB fluid, and sonographic Murphy sign. Moreover, there have been reported refinements in the use of US to evaluate patients with right upper quadrant pain and suspected AC. Wertz et al. [20] reported the transverse dimension of the GB more than 4 cm was found in 59% of their 60 patients with AC. Perez et al. [15] found that a cystic artery velocity of more than 40 cm/s had a high specificity of 94% for AC. However, the results were still inconclusive and needed further investigation.

This study has several limitations. First, a high risk of bias and applicability concerns in patient selection existed in more than one-fourth of the studies, limiting the generalizability. However, our study is by far the most comprehensive systematic review regarding US for the diagnosis of AC. Second, the majority of studies were conducted in Western countries. The results would be extrapolated cautiously to Asian patients. Third, the details of comorbidities and body mass indexes were lacking across the studies; thus, factors associated with false-negative and false-positive cases could not be thoroughly analyzed. Fourth, acalculous cholecystitis accounts for approximately 10% of patients with AC [53, 54]. However, AC was diagnosed in this review using criteria for the presence of gallstones. The extrapolation of the results should be cautioned for patients with acalculous cholecystitis. Last, patients have to fast for at least 6 h before US for a better illustration of the GB. However, most studies did not provide information on whether the patients were fasting or not. Also, ED patients would visit after a big meal. The diagnostic performance of US would be influenced by non-fasting patients.

## Conclusion

US is a good imaging modality for the diagnosis of AC with discriminative power. EP-performed US has a similar diagnostic performance to those by radiologists. Further investigations would be needed for the impact of US on the clinical management process and patient-centered outcomes.

### Supplementary Information

**Additional file 1**: Fig. S1 The summary receiver operating characteristic (SROC) curve of the included studies.

**Additional file 2**: Fig. S2 The forest plot of diagnostic performance of ultrasound performed by emergency physicians.

**Additional file 3**: Fig. S3 The forest plot of diagnostic performance of ultrasound by surgeons.

**Additional file 4**: Fig. S4 The forest plot of diagnostic performance of ultrasound by radiologists.

**Additional file 5**: Table S1 The complete literature search strategy.

## Data Availability

All data analyzed during this study are included in this published article.

## Abbreviations

AC: Acute cholecystitis
ED: Emergency department
GB: Gallbladder
US: Ultrasound
CT: Computed tomography
HIDA: Hepatobiliary iminodiacetic acid
EP: Emergency physician
PRISMA-DTA: Preferred Reporting Items for a Systematic Review and Meta-analysis of Diagnostic Test Accuracy Studies
QUADAS-2: Quality Assessment of Diagnostic Accuracy Studies-2
CI: Confidence interval
SROC: Summary receiver operating characteristics
PLR: Positive likelihood ratio
NLR: Negative likelihood ratio
