# Supplementary figures and images for: Diagnostic performance of ultrasound in acute cholecystitis: a systematic review and meta-analysis
Source: World J Emerg Surg. 2023 Nov 30;18:54. doi: 10.1186/s13017-023-00524-5 (PMC10687940; doi:10.1186/s13017-023-00524-5)

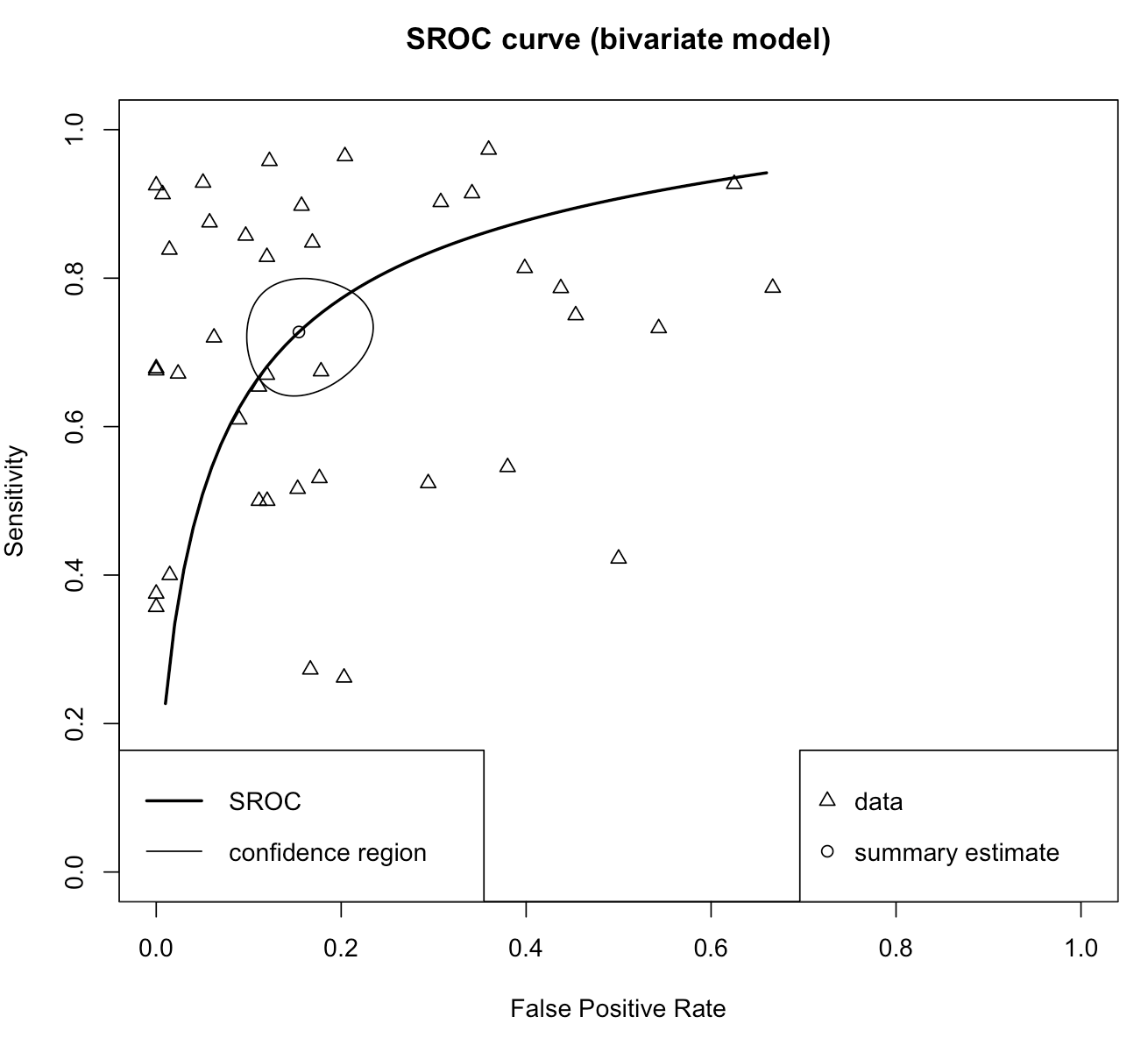

Supplement: Supplementary file 1 — Additional file 1: Fig. S1 The summary receiver operating characteristic (SROC) curve of the included studies. [file 13017_2023_524_MOESM1_ESM.tiff]

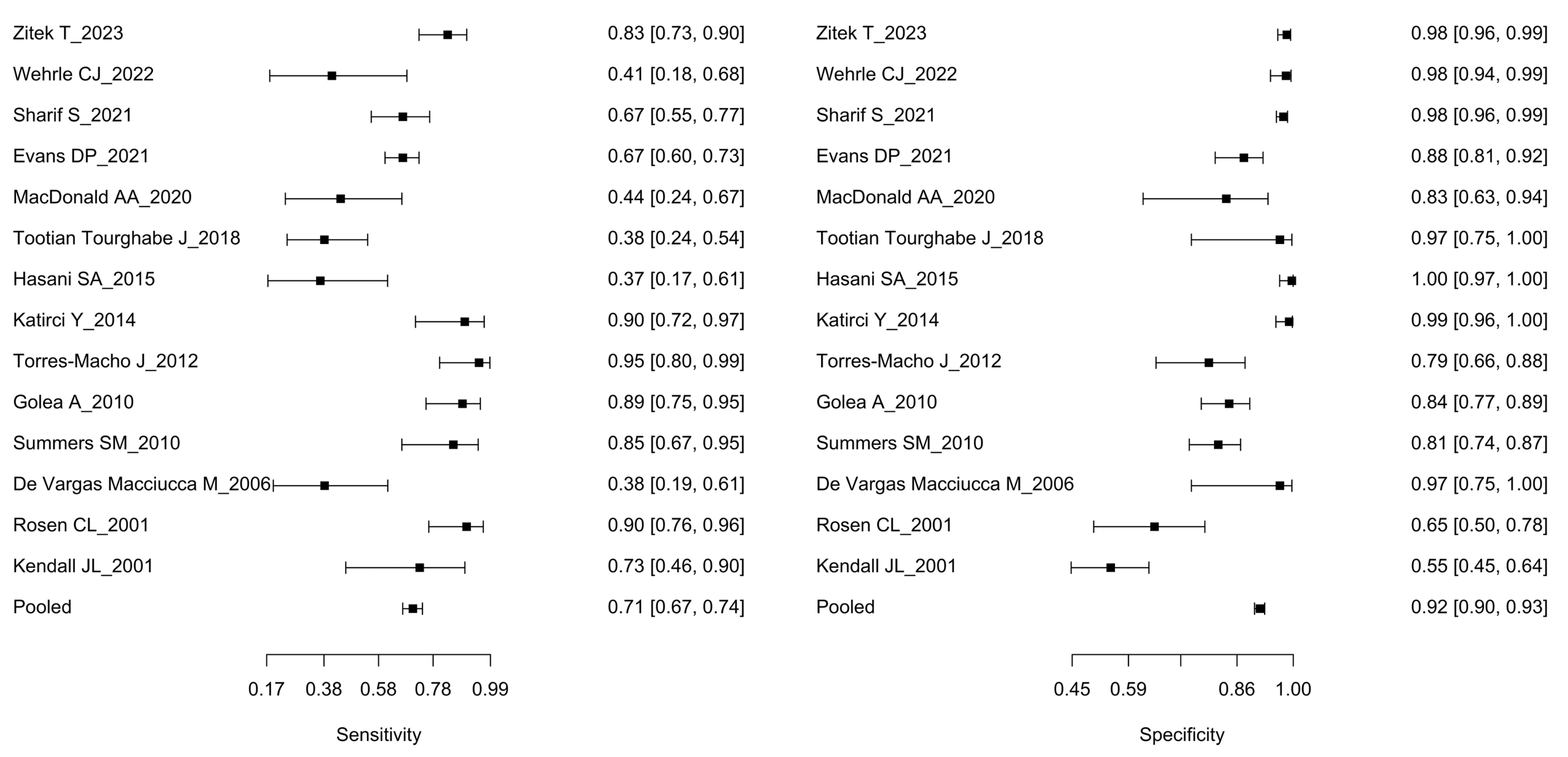

Supplement: Supplementary file 2 — Additional file 2: Fig. S2 The forest plot of diagnostic performance of ultrasound performed by emergency physicians. [file 13017_2023_524_MOESM2_ESM.tiff]

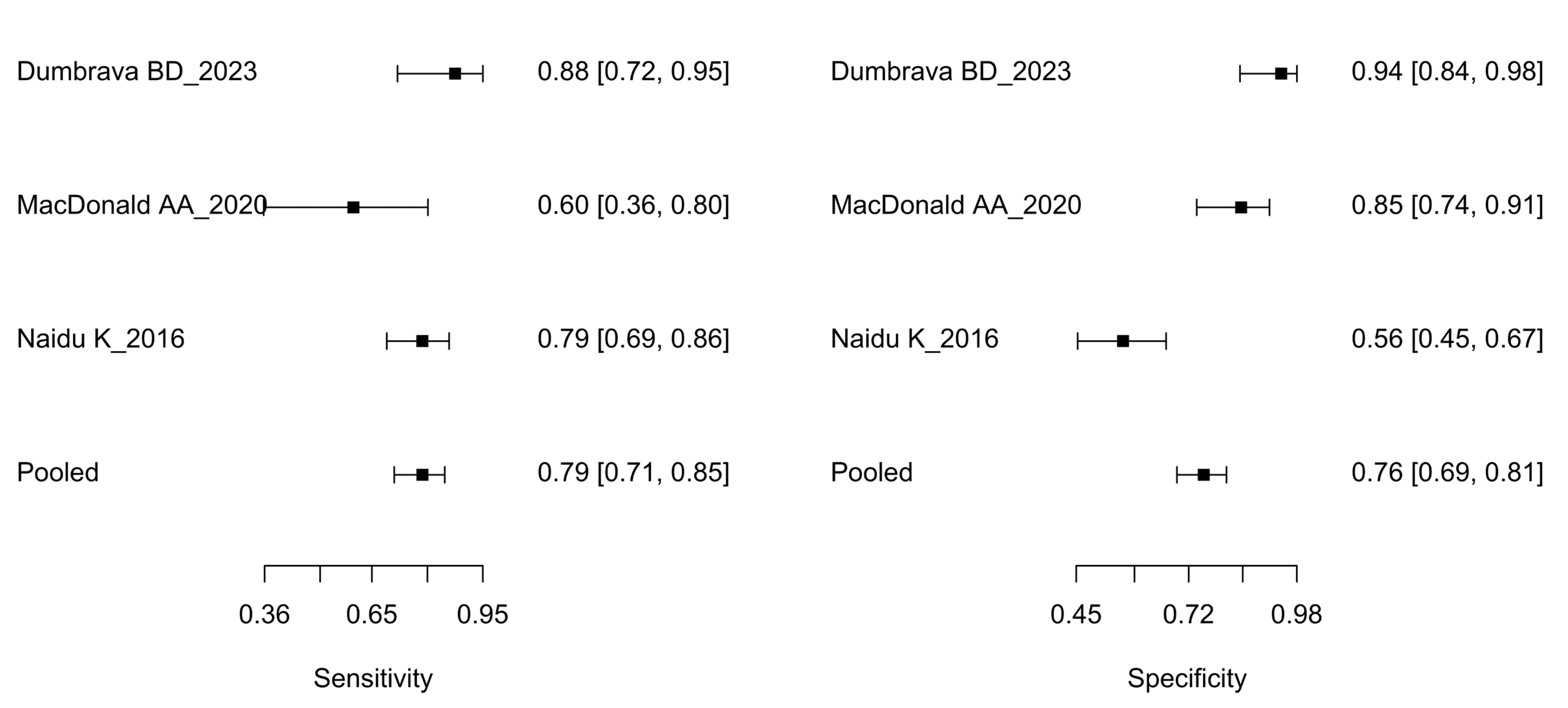

Supplement: Supplementary file 3 — Additional file 3: Fig. S3 The forest plot of diagnostic performance of ultrasound by surgeons. [file 13017_2023_524_MOESM3_ESM.tiff]

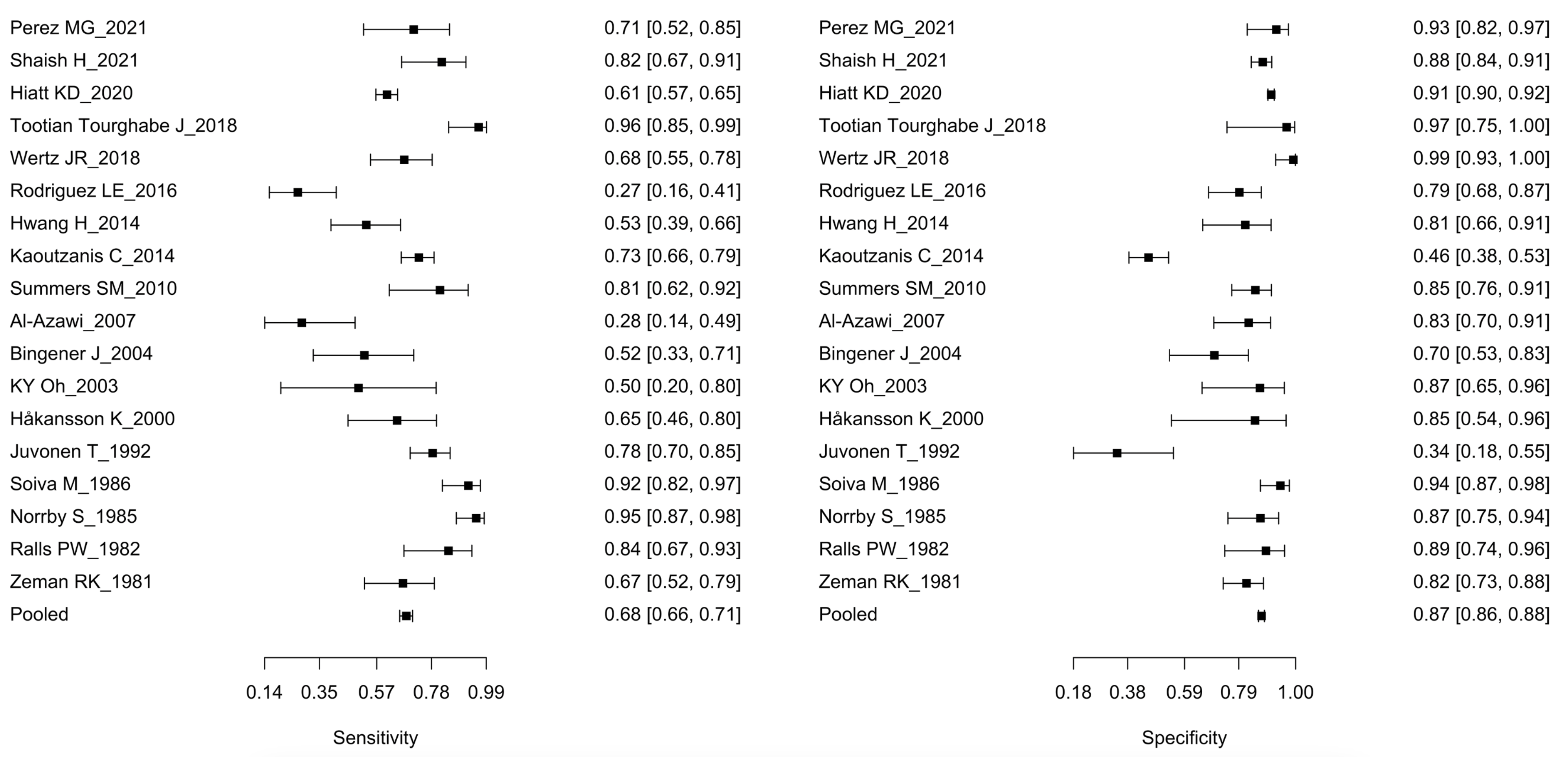

Supplement: Supplementary file 4 — Additional file 4: Fig. S4 The forest plot of diagnostic performance of ultrasound by radiologists. [file 13017_2023_524_MOESM4_ESM.tiff]
