# Supplementary material for: Diagnostic performance of ultrasound in acute cholecystitis: a systematic review and meta-analysis
Source: World J Emerg Surg. 2023 Nov 30;18:54. doi: 10.1186/s13017-023-00524-5 (PMC10687940; doi:10.1186/s13017-023-00524-5)
Supplement: Supplementary file 5 — Additional file 5: Table S1 The complete literature search strategy. [file 13017_2023_524_MOESM5_ESM.docx]

Supplementary Table 1. The complete literature search strategy.

| Criterion | Detail |
| --- | --- |
| Search terms | (Point-of-care ultrasound) OR (bedside ultrasound) OR (PoCUS) OR (emergency ultrasound) AND (acute cholecystitis) |
| Language | No restrictions |
| Timeframe | From inception to August 2023 |
| Database | MEDLINE, Embase, Cochrane Library |
| Inclusion criteria | Articles investigating the diagnostic performance of ultrasound for acute cholecystitis. |
